# The Task of Social Hygiene

**Published:** 1912-09-14

**Authors:** 


### THE TASK OF SOCIAL HYGIENE.*

### Havelock Ellis on the Health of Society.

As all competent readers of Havelock Ellis are aware, there is hardly a serious topic of controversy with which this great sociologist has not concerned himself in the domain which he, alone of English writers, has made pre-eminently his own, and which for short may be called Social Hygiene. His latest work is an epitome of many studies. The specialist in varied fields leaves his researches and sums up in a new volume their results for practical statesmanship. To the question, What are the tasks of civilisation at the opening of the twentieth century? the chapters of this volume give detailed replies: to define the status of women in the commonwealth, now in recognised process of change; to understand and profit by the significance of a falling birth-rate; to carry the stage of social reform a step further by undertaking some form of eugenics; to decide how far the unescapable questions of religion and sex instruction shall enter into education; to adjust criminal law to the distinction between immorality and crime, making the strength of the social reaction the test of crime where an anti-social act has been committed, and, where it has not, remembering that a disgusting person is not necessarily a criminal person; to compel nations to adjust their differences before an international tribunal with power to enforce its demands, as individuals and corporations have already been compelled to adjust them; to supply the world's urgent need for an auxiliary international language; and to give complementary play to the equally insistent claims of individualism and socialism.

Such is the matter of this book, which has the benefit characteristic of this author's work of being written from the European and not the insular point of vantage. The controversy with which the subjects are surrounded disperse like mists before the patient logic with which they are approached, as we learn that dogmatic religion should not be taught to children before puberty; that an international language must in practice be an artificial product when we come to examine the claims of existing languages to-day; that the growing weight of such stern forces as economic pressure and the cost of victory are making war a gradually more impossible task, and so forth.

### The Meaning of a Falling Birth-rate.

The value of the work, however, is not only in the conclusions at which it arrives, most of which among persons of intellectual distinction and authority will not be disputed, but in the process by which they are arrived at; yet, as to give this would be to rewrite the whole work, the two most controversial questions may be taken as examples of the author's logic: the birth-rate and eugenics. After pointing out the phenomenal expansion of the people of these islands during the early part of the nineteenth century, the writer notes the decline that has now become a steady process, shared by practically all other nations except Russia. He then forcibly reminds us that "a high or a low birth-rate has no meaning, so far as the growth of a nation is concerned, unless it is considered in relation to its death-rate," for "the natural increase of a nation is not the result of its birth-rate, but of its birth-rate minus its death-rate." He next proceeds to show that so far a high birth-rate and a high death-rate have always been found together, the reason being that large families produce parental carelessness, and that the net result in surviving children is relatively more than twice as great among women with families of three or four and those with eight or fifteen. This first part of the argument he sums up as follows: "A community which is reproducing itself rapidly must always be in an unstable state of disorganisation highly unfavourable to the welfare of its members . . . The high infantile mortality of the community with a high birth-rate merely means that that community is unconsciously making a violent and murderous effort to attain to the more stable and organised level of the country with a low birth-rate." This paves the way for a consideration of the significance of a falling birth-rate. Chief causes are voluntary restriction "due to the growth of intelligence, knowledge, and foresight"; delayed marriage; and the pathological sterility that always accompanies an increasingly urban life.

A high birth-rate and a high degree of prosperity are not concomitants; indeed "in so far as high fertility is associated with prosperity, it is with the prosperity of a young and unstable community, which has experienced a sudden increase of wealth and a sudden expansion," as the case of Western Australia serves to show. The result is that the restraints of foresight are temporarily removed. Beveridge has shown that "the years when the people of Great Britain marry most are the years when they drink most"—a significant fact which drives this argument home. The foresight which reduces the nation's drink-bill and marriage-rate is sometimes called selfishness by silly people who think foresight is not a virtue when you give it a bad name; but their position can be turned with ridiculous ease by the reminder that the altruistic parent who produced children in large numbers had forcibly to be restrained by Factory Acts from working them to death to increase the family—that is the parent's—income. Again, "if a high birth-rate were a mark of advanced civilisation we should expect to find it among the better social class of a community. But the reverse is the case." Diminished offspring is the response to improved environment. Zoological evolution is the process of substituting for large numbers of offspring great care in the nurture of a few.

### "The Essence of Civilisation."

A falling birth-rate is therefore seen to be of the essence of civilisation. In this connection let us add

* *The Task of Social Hygiene.* By Havelock Ellis. Constable and Co. 8s. 6d. net.

a most illuminating remark from another chapter: "The fighting instinct, and even the ruling spirit, are not synonymous with civilisation. The 'decline and fall' of empires by no means necessarily involves the decay of civilisation. It is now generally realised that the later Roman Empire was not, as was once thought, an age of social and moral degeneration. The State indeed was dissolving, but the individual was evolving." That is a summary of the argument with which Havelock Ellis meets the alarmists who lament so loudly the declining birth-rate. As the most controversial argument in the book, it best illustrates perhaps the force and matter of the volume. Against his contentions only one reply readily suggests itself—that, namely, the combination of the high death-rate with the high birth-rate is not in the nature of things, and that the lessons of the past belie the future in this matter. This, however, is merely speculation, and the closer the subject is studied the more reasons appear why a high death-rate should inevitably accompany a high birth-rate, whether considered from the historical or zoological side.

Space only remains to add that the chapter on Eugenics uses that term in the very widest sense as the science of all the agencies for the better breeding of the human race. He believes that "human breeding must proceed from impulses that arise voluntarily in human brains and wills," and is very justly, like Galton, not anxious to invoke legislation. This is sensible enough, but in his desire to win public sentiment over to his side we think he insists too strongly that "it would clearly be undesirable to breed men as animals are bred," for this seems to involve an exclusion of any form of experimental mating. He would have been wiser to supplement his caution that legislation is to be avoided by reminding his readers of the old classical respect for the impersonal sanctities of marriage, which placed it above personal wishes or preferences; of the still surviving example of this by which kings and queens marry for dynastic and not personal reasons; and to insist with greater emphasis than he does that all marriage involving reproduction is far more the community's than the lovers' affair. If, as he says, the day may soon come when men shall make themselves eunuchs for the Kingdom of Heaven's sake, the day may surely come when men and women shall mate themselves for the same reason. No one wishes to hurry them into it, but all who appreciate the importance of social hygiene must see the need for reminding the public that, far from being a natural right, the uncontrolled choice of marriage partners is only a late fashion of the past fifty years. Once that be grasped, the community's claim to register and control vitally all unions where children are involved is seen to be not merely a reasonable one, but an actual and living tradition.

